# The potential role of HGF-MET signaling and autophagy in the war of Alectinib versus Crizotinib against ALK-positive NSCLC

**DOI:** 10.1186/s13046-018-0707-5

**Published:** 2018-02-20

**Authors:** Xing Huang

**Affiliations:** 1 Key Laboratory of Precision Diagnosis and Treatment for Hepatobiliary and Pancreatic Tumor of Zhejiang Province, First Affiliated Hospital, School of Medicine, Zhejiang University, 79 Qingchun Road, Zhejiang, Hangzhou 310003 China; 2 The Key Laboratory of Developmental Genes and Human Disease, Institute of Life Sciences, Southeast University, Nanjing, Jiangsu 210096 China; 3 The Therapeutic Antibody Research Center of SEU-Alphamab, Southeast University, Nanjing, Jiangsu 210096 China

**Keywords:** NSCLC, ALK-targeted therapy, Alectinib, Crizotinib, HGF-MET signaling, Therapeutic resistance, Autophagy, LIR motif, Combined treatment

## Abstract

Non-small-cell lung cancer (NSCLC) is currently the leading cause of cancer-related death. Accumulating evidences suggest that overcoming the therapeutic resistance in NSCLC is a big challenge. Recently, the outcomes of two independent phase 3 trials regarding Alectinib versus Crizotinib in ALK-positive NSCLC are encouraging. However, given the potential relevance of HGF-MET signaling and especially autophagy to the war against ALK-positive NSCLC between Alectinib and Crizotinib, it’s too early to reach a convincing conclusion. Therefore, to further improve the therapeutic efficacy of ALK-positive NSCLC, this commentary highlights the negligence in design of relevant clinical trials, emphasizes the importance of molecular characteristics investigation, and discusses the prospect of combination therapy.

## Background

Non-small-cell lung cancer (NSCLC) is currently one of the leading cause of cancer-related death. Accumulating evidences suggest that overcoming the therapeutic resistance in NSCLC is a big challenge. Recently, two independent phase 3 trials regarding Alectinib versus Crizotinib in ALK-positive NSCLC, have been individually reported by Solange Peters et al. [1] in *NEJM* and Toyoaki Hida et al. [2] in *Lancet*. The outcomes are encouraging, and I appreciate the concerted effort for improving therapeutic efficacy in NSCLC. However, after careful analysis and consideration, I think it’s too early to reach a conclusion, because the both trials utterly neglect several key points and challenges that are highlighted as follows.

## Main text

First, Crizotinib is actually an ALK/MET double-targeted small molecule inhibitor. Hepatocyte growth factor (HGF) and its physiological receptor tyrosine kinase MET (HGFR) have been reported involved in almost all the aspects of cancer progression, including cancer development, growth, invasion and metastasis. HGF-MET signaling activates AKT- and ERK-mediated cancer cell survival, cycle, proliferation, motility and transformation. The significance of HGF-MET signaling makes them become critical targets in cancer therapy [3]. Unexpectedly, besides cancer cells themselves, MET also regulates cancer immune microenvironment. For instance, MET is required for recruitment and infiltration of anti-tumor neutrophil to abate metastasis [4]. The paradoxical effects of MET ought to force us to re-investigate whether it is really an applicable target in cancer therapy. Therefore, the states of MET and its ligand HGF need to be taken into consideration in the design and analysis of clinical trials. Alectinib combined with MET-targeted inhibitor versus Crizotinib is a more reasonable comparison.

Second, Crizotinib is able to induce autophagy in NSCLC [5]. Autophagy is a protective mechanism that is usually linked to cancer metabolic stress and therapeutic resistance, mainly on “self-eating” to recycle obsolete components to maintain necessary biogenesis for cancer survival. In fact, combined treatment with ALK inhibitor and autophagy blocker, like Chloroquine (CQ) or 3-Methyladenine (3-MA), can dramatically suppress cancer cell viability and colony formation in many kinds of NSCLC [6]. This indicates autophagy plays a pivotal role in ALK-driven onco-signalings, and autophagic state is likely to determine ALK-targeted therapeutic efficacy in NSCLC.

Furthermore, ALK contains the typical LC3-interacting region (LIR) motif. LIR motif is the principal structural foundation for autophagy machinery [7]. By virtue of bioinformatics analysis, seventeen various types of LIR motifs are identified in MAM1, MAM2, TM, PTK domains or other regions of ALK (Table 1), which means there may be a direct interplay between ALK and autophagy. Of note, some of LIR motifs locate in kinase activity center of ALK. Given that de-phosphorylation in LIR motif has been proved capable to enhance its interaction with LC3 [8], a crucial issue arises: should we just focus on inhibiting the kinase activation in ALK-targeted therapy for NSCLC? Because if only targeting kinase activity, ALK may immediately turn to induce autophagy for resisting cancer therapeutic stress via its inactivation-caused de-phosphorylated LIR motif. It seems like “when one door shuts, another opens”, and will be a big challenge for all the ALK-targeted therapies (including but not limited to NSCLC).

**Table 1:** Potential LIR Motif in ALK

No	Position	Domain	Type	Sequence
1	46–49	Extracellular	Y	YSRL
2	253–256	Extracellular	F	FPFL
3	290–293	MAM1	W	WRRI
4	320–323	MAM1	F	FLLL
5	386–389	MAM1	W	WTVL
6	419–422	MAM1	F	FFAL
7	587–590	MAM2	Y	YEGL
8	595–598	MAM2	W	WMVL
9	625–628	MAM2	F	FDNI
10	635–638	MAM2	Y	YLTI
11	772–775	Extracellular	Y	YILV
12	1052–1055	TM	F	FSGI
13	1127–1130	PTK	F	FGEV
14	1193–1196	PTK	F	FILL
15	1315–1318	PTK	F	FGVL
16	1376–1379	PTK	F	FAII
17	1401–1404	Intracellular	Y	YGPL

In addition, as one coin has two sides, emerging evidences suggest that autophagy has mutually contradictory effects on cancer [9]. For example, cancer cell autonomous autophagy is required for chemotherapy-induced immune surveillance by releasing ATP to activate immunocytes, whereas surrounding normal cells-generated micro-environmental autophagy promotes tumor growth by supplementing abundant extra-metabolites. Moreover, autophagy has distinct phase-dependent functions in cancer progression, for example, preventing malignant transformation, but favoring growth and metastasis [10]. Thus, it’s in dire need to divide ALK-positive NSCLC into molecular characteristics of HGF-MET signaling or / and autophagy-based sub-types for recruitment of the most eligible patients.

## Conclusions

Taken together, even the both trials provide strong evidences supporting the superiority and safety of Alectinib in comparison with Crizotinib in ALK-positive NSCLC, I still strongly suggest authors further investigate potential roles of HGF-MET signaling and autophagy in determining sensitivity or resistance to ALK-targeted therapy for NSCLC. On the other side, since the anti-cancer effects and safety of autophagy blocker have been testified in several cancer therapies, ALK-autophagy double-targeted strategy maybe a simple and practicable method to improve NSCLC therapeutic efficiency in the future.

## Abbreviations

3-MA: 3-Methyladenine
CQ: Chloroquine
HGF: Hepatocyte growth factor
LIR: LC3-interacting region
NSCLC: Non-small-cell lung cancer
